# Diffusion Tensor Imaging and Resting-State Functional MRI-Scanning in 5- and 6-Year-Old Children: Training Protocol and Motion Assessment

**DOI:** 10.1371/journal.pone.0094019

**Published:** 2014-04-09

**Authors:** Catherine Theys, Jan Wouters, Pol Ghesquière

**Affiliations:** 1 ExpORL, KU Leuven, Leuven, Belgium; 2 New Zealand Institute of Language, Brain and Behaviour, University of Canterbury, Christchurch, New Zealand; 3 Parenting and Special Education Research Unit, KU Leuven, Leuven, Belgium; Lyon Neuroscience Research Center, France

## Abstract

Advanced Magnetic Resonance Imaging (MRI) techniques such as Diffusion Tensor Imaging (DTI) and resting-state functional MRI (rfMRI) are widely used to study structural and functional neural connectivity. However, as these techniques are highly sensitive to motion artifacts and require a considerable amount of time for image acquisition, successful acquisition of these images can be challenging to complete with certain populations. This is especially true for young children. This paper describes a new approach termed the ‘submarine protocol’, designed to prepare 5- and 6-year-old children for advanced MRI scanning. The submarine protocol aims to ensure that successful scans can be acquired in a time- and resource-efficient manner, without the need for sedation. This manuscript outlines the protocol and details its outcomes, as measured through the number of children who completed the scanning procedure and analysis of the degree of motion present in the acquired images. Seventy-six children aged between 5.8 and 6.9 years were trained using the submarine protocol and subsequently underwent DTI and rfMRI scanning. After completing the submarine protocol, 75 of the 76 children (99%) completed their DTI-scan and 72 children (95%) completed the full 35-minute scan session. Results of diffusion data, acquired in 75 children, showed that the motion in 60 of the scans (80%) did not exceed the threshold for excessive motion. In the rfMRI scans, this was the case for 62 of the 71 scans (87%). When placed in the context of previous studies, the motion data of the 5- and 6-year-old children reported here were as good as, or better than those previously reported for groups of older children (i.e., 8-year-olds). Overall, this study shows that the submarine protocol can be used successfully to acquire DTI and rfMRI scans in 5 and 6-year-old children, without the need for sedation or lengthy training procedures.

## Introduction

Advanced Magnetic Resonance Imaging (MRI) techniques such as Diffusion Tensor Imaging (DTI) and resting-state functional MRI (rfMRI) are widely used to study structural and functional neural connectivity. However, as these techniques are highly sensitive to motion artifacts [1]–[4] and require a considerable amount of time for image acquisition, successful acquisition of these images can be challenging to complete with certain populations. This is especially true for young children. Hence, studies using DTI and rfMRI to assess neural connectivity have mainly focused on the adult and adolescent population [3], [5]–[7]. However, there are numerous developmental disorders (e.g., dyslexia, stuttering and autism spectrum disorders) for which the acquisition of images related to structural and functional neural connectivity in younger children is valuable. Without such data, neural changes observed in adolescents and adults cannot unequivocally be identified as causal mechanisms due to influences such as compensatory processes and medication that may have altered connectivity patterns over time. Therefore, despite its challenges, undertaking advanced MR imaging in young children is important to advance our knowledge of the neural mechanisms at play.

For conventional structural MRI, research has described different techniques to restrict children's motion and to increase their compliance with the scanning procedures. These include behavioral training, training sessions in a mock scanner, and the use of natural sleep or sedation [8]. Sedation, in particular, enables clinicians to bypass potential problems with cooperation, ensuring good image quality [9], [10]. However, it is not ethically acceptable to sedate children for research purposes, especially because sedation includes potential risks for the child [8]. In addition, sedating children is a costly, time-consuming process that prohibits the active participation from the child required for the acquisition of functional MRI (fMRI) scans and interferes with the BOLD response [11]. An excellent review of the few structural and functional MRI studies conducted to date using non-sedated children is provided by Raschle and colleagues [12]. In these studies, children are commonly trained in a mock scanner a few days to weeks before the scanning session [8], [13]–[15]. Such training sessions usually require the child to visit the hospital on several occasions [12], [14] and may increase anxiety levels in some children [16]. Given the limitations of this approach, we aimed to develop a training protocol for 5- and 6-year-old children that could be implemented in a single scanning visit (i.e., did not require attendance at one or more pre-scanning sessions) and did not require the use of a mock scanner.

Any neuroimaging study aims to achieve a high success rate and excellent scan quality. However, descriptions of scanning procedures and their resultant outcomes for young children are sparse. For conventional structural MRI-scanning and task-based fMRI scanning, the reported success rates of scanning such young children, while they are awake, vary widely [12]. This is due to considerable variability in methods and criteria used [12], [15], [17]. Success rates themselves also vary in their calculation. One common approach has been to describe the number of children completing the full scan battery or part thereof. For example, Weber Byars and colleagues reported that 9 of 21 (43%) 5-year-olds and 8 of 15 (53%) 6-year-old children completed at least 1 fMRI run after viewing a video and receiving a tour of the MRI environment before their scan session [17]. In another study, children received a training session in a mock scanner/tunnel and were exposed to scanner sounds to prepare them for their scan. Following this training session, 12 of 22 (55%) typically developing 4- to 6-year-old children completed 2 fMRI runs [16]. A significantly higher success rate was demonstrated by Raschle and colleagues, who found that 44 of 45 (98%) 4-6 year-old children successfully completed functional MRI scans after being trained in a mock scanner [12]. These studies have shown that anatomical and task-based MRI scanning of young children is possible, albeit with varying success rates. However, reports of the quality of the acquired images, in particular the amount of motion during the scan, are required to guide researchers and clinicians in their choice of procedures.

Thus far, few studies have provided direct information on the motion present in the scans in 5- and 6-year-old children. These have mainly addressed the outcomes of conventional structural MRI and task-based fMRI scans. To our knowledge, specific reports detailing the observed motion in individual children have not been reported previously for advanced rfMRI and DTI scanning in this age group. Studies reporting conventional structural MRI scans have focused on whether a scan was diagnostic or non-diagnostic based on the presence of motion artifacts in the images. For example, in a study of 155 children under 6 years of age, 117 MRI scans (75%) were labeled as diagnostic [8], as were 53 of 60 MRI scans (83%) acquired in a study including children under 7 years of age [14]. However, in both studies, the ‘diagnostic’ label was difficult to interpret as it included scans with varying degrees of motion (from no motion to moderate motion artifacts). Other studies, focusing on task-based functional MRI scans, have used more objective measures of analysis – in this case the amount of motion present between different acquired volumes. Where reported, the maximum motion deemed acceptable in studies with 5- and 6-year-old children is often limited to the size of one voxel [14], although some studies have included scans exceeding this threshold [16], [18]. In the task-related fMRI study of de Bie and colleagues, 23 of 36 children (64%) under 7 years of age had less than 3 mm maximum motion during two runs of the fMRI scan in any plane [14]. Klaver and colleagues reported that 1 of the 10 children in their study (5.6–6.9 years) showed 3.5 mm movement in one direction, while all the other children had less than 2.5 mm movement in any plane during their fMRI scans [18].

For advanced rfMRI and DTI imaging, detailed assessments of the motion present in 5- and 6-year-old children are lacking. Specifically for rfMRI, a number of studies have recently highlighted the influence of motion on the outcomes of rfMRI scans in adolescents and adults. Unfortunately, due to different methods used for analyzing and reporting motion, comparison of the data across studies is difficult. For example, motion parameters have been summarized as mean motion or maximum motion and have been calculated for each plane separately, were based on a summary statistic of the three translation parameters, or were based on a combination of the translation and rotation parameters [1]–[3], [15], [19], [20]. In a sample of 1000 healthy adults, mean relative volume-to-volume displacement, based on the 3 translations, was 0.05±0.004 mm. In this study, 8.5% of the subjects were considered outliers and 2.8% were considered extreme outliers as their mean motion was greater than 2.0 and 2.5 standard deviations from the mean, respectively [1]. Also calculating relative volume-to-volume displacement, Satterthwaite and colleagues reported 0.14±0.23 mm mean relative displacement in their sample of 456 adolescents (15.6±3.4 years). After exclusion of 35 subjects with gross head motion (defined as >0.55 mm mean displacement), the overall mean relative displacement was 0.09±0.09 mm [3]. Power and colleagues reported mean root mean squared movement (RMS, of translation and rotation parameters) of 0.51±0.29 mm and 0.70±0.31 mm motion in a dataset of 22 children (8.5±1 years), and 42 children (8.8±0.7 years), respectively after exclusion of subjects with mean RMS movement exceeding 1.5 mm (half of a voxel's size) [2]. Another recent study reported rfMRI data of 21 children (12.5±2.2 years) without rejecting datasets due to excessive motion. For these subjects, the median total displacement over time (based on translation and rotation parameters) was 0.47 mm (range 0.08–8.1 mm) [19]. In a research paper reporting rfMRI data in children in our target age group (5.1–8.1 years), 18 of 23 children (78%) exhibited less than 4 mm maximum movement. It is unclear if the assessment of motion included both rotations and translations [15].

In comparison with rfMRI, the influence of motion on structural connectivity as measured with DTI is still poorly understood [4] and specific assessment of individual movement data in 5- and 6-year old children is absent in the DTI literature. In a study focusing on motion in a group of 49 healthy adults, Ling and colleagues reported that the mean relative translational motion ranged from 0.13±0.03 mm to 0.30±0.16 mm and the mean relative rotation varied from 0.08±0.02 to 0.14±0.04 degrees over the 3 different axis. Head motion in their subjects showed a positive correlation with differences in fractional anisotropy and mean diffusivity. Despite these influences of motion on the quality of the DTI images, reports of motion present in the images or the criteria used for considering image quality as insufficient are often not reported in DTI studies (but see [21]).

The present study aims to give a detailed description of a protocol developed to prepare 5- and 6-year-old children for their DTI and rfMRI scans. This training protocol was designed to allow time and resource efficient scanning without the use of a mock scanner. By providing detailed information on the motion present in the DTI and rfMRI scans, we aim to inform researchers and clinicians of the results that can be expected when using the described protocol (i.e., in terms of completion rate of the scans and motion present in the data). We anticipate that this information will enhance researcher's ability to make informed decisions on study sample size and feasibility using advanced MRI techniques.

## Methods

### 1. Subjects

Using the submarine protocol described in section 3.1, 76 consecutive children were prepared for scanning for research purposes. All participating children were typically developing and aged between 5.8 and 6.9 years (mean 6.2 years). Forty participants had at least one close relative with dyslexia and were part of a high-risk group for the development of dyslexia. The remaining 36 children formed an age- and gender-matched control group. Forty-six of the children were boys.

### 2. Ethics statement

This study was conducted in accordance with the declaration of Helsinki and was approved by the ethical committee of the University Hospitals Leuven. Parents provided written informed consent and the children assented verbally to participate in the study.

### 3. Procedure

#### 3.1 ‘Submarine’ protocol

The overall aim of the protocol was to make the MRI scanning session a pleasant experience for the children by immersing them in a story about a submarine adventure. Furthermore, the protocol was designed to achieve this in a time and resource efficient manner, without lengthy preparation procedures or repeated visits to the hospital.

The protocol consisted of three phases: (i) initial contact phase, (ii) pre-scanning preparation phase, and (iii) MRI scan session. A summary of the submarine protocol is provided below, with a step-by-step description provided in the Text S1.

##### Phase 1: Initial contact phase

Contact was established with the parents prior to the hospital visit. During this phase, we aimed to fully inform parents about the purpose of the scans and detail the practical aspects of an MRI scanning session. This information was also sent by email and included a link to two movies. The first movie was tailored to parents. It showed the procedure, acquired images and safety procedures applied during a scan session with a 5-year-old. The second movie served as an introduction video for children, featuring Whally the Whale. Whally the Whale is a stuffed toy that plays a lead role throughout the protocol, guiding the child through all the steps of the upcoming hospital visit.

##### Phase 2: Pre-scanning preparation phase

The second phase consisted of a 45 minute session in which the child completed 6 tasks. Each of these tasks allowed the child to become familiarized with the potentially difficult aspects of undergoing an MRI scan – restricted movement, wearing earplugs and headphones to reduce noise levels, going through a small tunnel, trusting the researcher and wearing a head coil. The six pre-scanning preparation tasks were as follows:

1. *Popping bubbles*: An icebreaker task designed to make the child feel at ease with the researcher. It involved blowing and popping bubbles together.
2. *Good and blurry pictures*: This task was used to explain to the child that the pictures of his/her brain will be blurry if he/she moves while lying in the scanner. It involved identifying sharp and blurry pictures taken by Whally the Whale.
3. *Picture frame*: The child was provided with a picture frame to decorate, and the child was informed that the picture frame would ultimately house a picture of his/her brain. This task was employed to maximize a child's cooperation in the scanner through the visualization of a reward.
4. *Candy on nose*: The child learned how to lie still while balancing candy on his/her nose.
5. *Bucket talk*: A colorful bucket with a face drawn on it was placed over the child's head to help him/her get used to the feeling of wearing a ‘helmet’ and answering the researcher's questions without moving his/her head.
6. *Tunnel crawl*: The child wore ear plugs and headphones necessary to block the noise of the submarine and crawled through a small tunnel, wearing the hearing protection, to experience the feeling of being in a confined space.

Following successful completion of these tasks, the child earned the diploma of ‘submarine captain’. This honor enabled him/her to start the submarine journey.

##### Phase 3: MRI scan session

The actual scanning session lasted approximately 35 minutes. It started by introducing the child to the scanner room. This room was decorated in a submarine theme by placing a large cardboard submarine in front of the scanner and hiding all medical appliances behind colorful fishes, shells and seaweed. Before and during the scan, the researcher informed the child again of all the steps about to occur. For example, the submarine will ‘dive’ multiple times and each time it dives, the engine noise will change a little and you may feel the vibrations of the engine. Although the children understood that they would not dive in an actual submarine, description of what was about to happen using terminology related to the submarine theme allowed them to perceive these noises and vibrations as a normal part of the process. We recommend that throughout the 3 phases of the protocol, the same person should be involved. That is, a single person should initiate the contact with the parents, feature in the videos, prepare the child for their scan, and communicate with the child while in the scanner. The enthusiasm of the researcher also plays a crucial role in keeping the child immersed in the submarine story.

#### 3.2 Data acquisition

The children underwent a Diffusion Tensor Imaging (DTI) scan, anatomical scan, and resting-state functional MRI (rfMRI) scan, respectively. They were scanned on a 3T Philips scanner (Best, The Netherlands) with a 32-channel head coil. The DTI data were acquired using an optimized single-shot spin-echo, echo planar imaging sequence with the following parameters: 58 contiguous sagittal slices, slice thickness  = 2.5 mm, repetition time (TR)  = 7.6 s, echo time (TE)  = 65 ms, field-of-view (FOV)  = 200×240 mm, acquisition time 10 min 32 s. Diffusion gradients were applied in 60 noncolinear directions with a b-value of 1300 s/mm^2^. Six nondiffusion-weighted images were acquired and summarized into one nondiffusion-weighted image. The anatomical MPRAGE was acquired with a FOV of 250×250 mm, 182 coronal slices and 1.2 mm slice thickness. The rfMRI data were acquired with the following parameters: 31 contiguous transversal slices, slice thickness  = 4 mm, TR  = 1.7 sec., TE = 33 ms, FOV = 230×230 mm, acquisition time 7 min 15 s. The total scanning time was 26 minutes. Pauses were included between the scans to interact with the child. Therefore, the total time spent in the scanner was approximately 35 min. The child watched a movie during all the scans with the exception of the rfMRI scan.

#### 3.3 Data analysis

##### DTI and rfMRI preprocessing

Preprocessing of the DTI data was performed using ExploreDTI [22]. During the motion and eddy current correction of the diffusion-weighted images, the b-matrix was corrected for the rotational component of subject motion to account for deviations in the diffusion weighting originating from these rotations (Robust EStimation of Tensors by Outlier REjection, RESTORE-approach, [23]). The rfMRI data were preprocessed using FSL (FMRIB's Software Library, www.fmrib.ox.ac.uk/fsl, [24]). Motion correction was performed using MCFLIRT [25].

##### Motion parameters

Head motion metrics were calculated to provide a quantitative measure of the outcome of the scans. They were derived from the 3 translation (x, y and z, in mm) and 3 rotation (α, β and γ, in radians) parameters calculated during the DTI and rfMRI preprocessing steps. To acquire a summary measure of motion, the root-mean-square of the 6 parameters describing the rigid body movement was calculated for each volume [2]. Similar to previous studies, rotational displacements were converted from radians to millimeters by calculating displacement on the surface of a sphere with a radius of 50 mm. This is approximately the mean distance from the cerebral cortex to the center of the head [2]. The one-dimensional motion timeseries can be calculated to measure the RMS displacement relative to a single reference volume (absolute displacement, RMS_abs_), or relative to the preceding volume (relative displacement, RMS_rel_ in absolute values). Both RMS_abs_ and RMS_rel_ will be reported, the latter to reduce the likelihood of inducing a bias based on a few large movements [4]. Mean RMS_abs_ over half of a voxel's width will be used as threshold for considering data useful [2], [26]. However, depending on the purpose of the study, different techniques for summarizing motion and different associated criteria for determining thresholds have been used previously. To allow comparison of our data with those of previous studies, we also report mean total and relative displacement for each of the 3 translations (in mm) and rotations (in degrees) for the DTI data [4]. For the rfMRI data, we also calculate mean relative displacement (MRD). This is calculated as the mean absolute displacement of each brain volume as compared to the previous volume, with displacement  =  square root (x^2^+y^2^+z^2^) [1].

##### Statistical analysis

Statistical analyses were performed using IBM SPSS Statistics 20. Due to the skewness of the motion data, the main results are summarized by reporting medians and range. Boxplots of the data for individual children are shown for visual representation of the data. However, to allow comparison of the results with previous studies, means and standard deviations are reported for sections of the results. For group comparisons of median RMS_abs_ the Mann-Whitney U-Test was used. The Wilcoxon Signed Ranks T-Test was calculated to compare the RMS_rel_ between the first and second halves of the rfMRI scans. For both tests, p≤.05 was used as the threshold for significance.

## Results

### 1. Completion rate of data acquisition

After training with the protocol, 75 of the 76 children (99%) were able to complete DTI scanning and 72 children (95%) completed the full 35-minute scan period. One of the 76 children was not scanned because she was afraid of confined spaces and refused to go into the scanner. One other child was afraid to enter the tunnel of the scanner during a first visit, but agreed to return a second time and was scanned successfully during that second session. All of the 75 children who entered the scanner completed the DTI scan, which was the first of the 3 longer scan sequences. In 3 children, the scan session was aborted before the anatomical and rfMRI scan because of excessive movement or the child's request to discontinue the scan.

### 2. Motion data

#### 2.1 DTI scans

**Figure 1:**
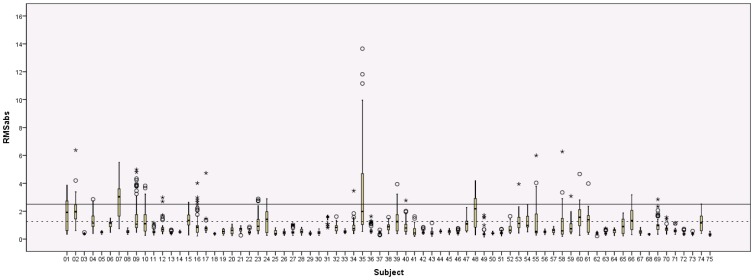
Boxplots of the RMS_abs_ displacement over the time-series of the DTI scans. Boxplots of the absolute root mean squared motion are shown for each of the 75 children that underwent DTI scanning. Continuous line  =  width of one voxel, dotted line  =  half a voxel's width.

The motion parameters for the 75 DTI scans are listed in Table S1 and visualized in Figure 1. The median RMS_abs_ displacement over time across the 75 children was 0.59 mm (range 0.31 to 3.04 mm). There was no significant difference in median RMS_abs_ between boys (median = 0.59 mm) and girls (median = 0.63 mm; U(73) = 668, p = .99). The maximum RMS_abs_ during the 10 min 32 s recording of the DTI-scan varied from 0.41 to 13.65 mm over the 75 children, with a median of 1.50 mm. The median RMS_rel_ displacement for the individual DTI scans varied from 0.03 mm to 0.61 mm over the 75 subjects, with a median of 0.07 mm.

The mean RMS_abs_ exceeded the threshold of 1.25 mm (half the voxel size) in 15 of the 75 subjects (20%). Similar to Ling and colleagues [4], the total and relative motion for each of the 6 translation and rotation parameters were calculated and subjects with more than 3 standard deviations total or relative motion in one of the planes were considered to be have extreme head motion. Eight of the 75 subjects (11%) in our sample exceeded this threshold for extreme motion. The total and relative motion after excluding these subjects is reported in Table S2.

#### 2.2 rfMRI-scans

**Figure 2:**
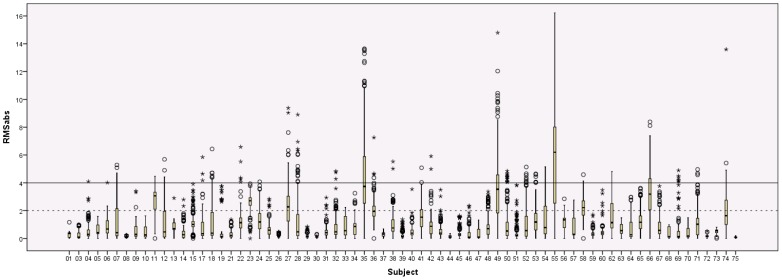
Boxplots of the RMS_abs_ displacement over the time-series of the rfMRI scans. Boxplots of the absolute root mean squared motion are shown for each of the 71 children that underwent rfMRI scanning. Continuous line  =  width of one voxel, dotted line  =  half a voxel's width.

**Figure 3:**
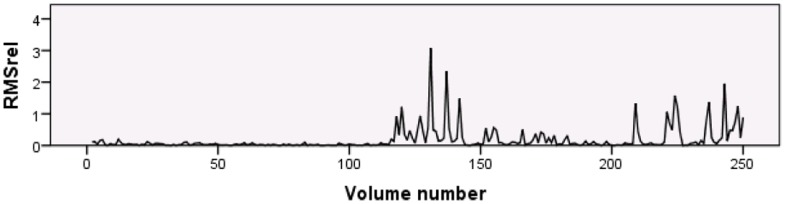
RMS_rel_ rfMRI time-series of subject 11. Subject 11 showed an obvious increase in motion during the second half of the rfMRI scan.

Seventy-two children underwent rfMRI scanning. The dataset of 1 of these children was incomplete due to technical problems. The motion metrics for remaining 71 individual children can be found in Table S3 and visualized in Figure 2. The median RMS_abs_ across subjects was 0.47 mm (range 0.07 to 6.20 mm). The maximum RMS_abs_ varied from 0.20 to 16.22 mm, with a median of 3.54 mm. The median RMS_rel_ over the scans of the 71 individual children varied from <0.01 mm to 0.35 mm, with a median of 0.03 mm. No statistically significant differences in median RMS_abs_ were present between boys (median = 0.47 mm) and girls (median 0.41 mm; U(69) = 538, p = .51). Visual inspection of the RMS_rel_ timeseries showed an apparent increase in motion during the second half of the scan for some of the subjects (see Figure 3 for an extreme example). Comparison of the median RMS_rel_ between the first 125 (median = 0.03 mm) and last 125 volumes (median = 0.04 mm) of each subject showed significantly more movement during the second half of the acquisition period (T(69) = 1903, p<.001).

In the present study, exclusion of subjects with a mean RMS_abs_ larger than 2 mm (half a voxel size) leads to the exclusion of 9 of the 71 (13%) rfMRI scans. For comparison of our data with the data of 8-year-old children reported by Power and colleagues [2], applying a threshold of 1.5 mm RMS_abs_ resulted in the exclusion of 12 of the 71 scans (17%), with the resulting 59 children displaying a mean RMS_abs_ motion of 0.64±0.37 mm. Using MRD, the mean motion in the scans of the 71 individual children varied from 0.03 mm to 2.03 mm (mean 0.32±0.36 mm). After exclusion of 10 subjects (14%) with mean MRD exceeding 0.55 mm [3], the summarized mean MRD over the remaining 62 subjects was 0.20±0.13 mm.

## Discussion

Scanning of children under 7 years of age without sedation is important for both clinical and research purposes. The present report aimed to provide a detailed description of a protocol for training 5- and 6-year-old children for advanced MR imaging. The submarine protocol was designed to allow scanning in a time and resource efficient manner without the need for a training session in a mock scanner. By immersing the children in a story about a submarine adventure, the children were prepared for the potentially difficult aspects of undergoing their MRI scans. To assess the success of the protocol, the completion rate of scan acquisition and a quantitative assessment of the motion data are reported for a group of 76 consecutive children prepared with this behavioral protocol.

### 1. Scan completion rates

DTI data were acquired in 75 of the 76 (99%) children and 72 (95%) of them underwent the full scan sequence, including the longer DTI, MPRAGE and rfMRI sequences. This is considerably higher compared to the previously reported completion rates of 43% and 53% in 5- and 6-year-olds prepared on the day of their scan without using a mock scanner [17]. Furthermore, the results show that our rates for completion of the scan protocol are among the most positive (ranging from 55%–98%) for scanning children in this age group following preparation with a mock scanner training session [8], [12], [16].

### 2. Motion during DTI scans

During the DTI scans, the data of 60 of the 75 children (80%) had a mean RMS_abs_ remaining under half a voxel's width thus remained under this threshold for excessive motion. It is evident that using other metrics, and thus other associated thresholds, may result in different outcomes. Using the metrics of Ling and colleagues, a smaller group of 8 of the 75 children (11%) displayed extreme movement [4]. In the adult study, 3 of 52 subjects (6%) were excluded based on this criterion. The higher exclusion rate in the present study is not surprising considering the large difference in age between the subjects in the studies. Comparison of the resulting data, after removal of the subjects with extreme movement, showed that the translation metrics of our young children were within the range of those reported in adults but that the children showed larger head rotations in comparison to this adult group. When focusing on maximum RMS_abs_, the motion in 49 of the 75 children (65%) did not exceed the width of one voxel. However, indices of maximum motion do not allow us to assess whether this maximum was due to a single larger movement during a scan with otherwise very limited movement or whether this amount of motion was present continuously during the scan. Although the detected maximum motion may be the same in scans with very limited movement and scans with continuous movement, obvious differences in the quality of the scans will be present. In the scan with a single larger movement only a small portion of the total number of diffusion weighted images may be corrupted in comparison with the scan with multiple larger movements [27]. Indeed, the boxplots in Figure 1 show that considerable variation existed in the movement patterns of the children. Overall, for the majority of children, there was very little movement throughout the scans with only a few instances of larger movement.

### 3. Motion during rfMRI scans

For the rfMRI data, the mean RMS_abs_ remained under 2 mm (half a voxel's width) for the scans of 62 of the 71 children (87%). Using this criterion, more rfMRI scans remained under the motion threshold compared to the DTI scans where a threshold of 1.25 mm was used (87% for the rfMRI versus 80% of the DTI scans). However, when focusing on maximum RMS (see Tables S1 and S3 and Figures 1 and 2), these values were larger in the rfMRI scans compared to the DTI scans. Based on our observation during the scans, this increase in movement may have been triggered by the fact that the child's movie was turned off. The observation that visual stimulation helps the children to restrict their head movements has been reported before [28]. Also, the increase in movement may be related to the rfMRI scan being the last scan in the session. We opted to keep the rfMRI acquisition as the last scan in the sequence because the DTI data were considered to be of primary importance in the study. Furthermore, we did not want to interrupt the children's movie for the lengthy rfMRI acquisition.

Comparison of the first and second half of the rfMRI scan showed that the children exhibited a significant increase in movement during the second half of the acquisition period. On the basis of this observation, it may be useful to include two shorter resting-state scans instead of one longer scan, allowing the researcher to remind the child to refrain from moving and to provide the necessary distraction between the scans. In addition to keeping the rfMRI scan time as short as possible, researchers might consider using an active fMRI task when scanning young children. In a previous study, most of the 12-year-old children showed larger maximum total displacement during rfMRI data acquisition compared to task-based fMRI [19]. This would allow the child to focus his/her attention on the task at hand, which may decrease excessive movements associated with boredom. When active participation in a functional task is required, it might be helpful to include a training session to practice the task in the preparation phase.

Based on measurements of MRD, the mean motion of 0.32±0.36 mm in our children was much higher compared to the MRD of 0.05±0.004 mm reported in healthy 20-year-old volunteers [1]. In this adult study, males moved significantly more than females, a difference that was not present in our younger population. The MRD data reported for adolescents (15.6±3.4 years), 0.14±0.23 mm, fall in between those of our young children and those of adults [3]. The subgroup of 8-year-old children trained with a mock scanner in the latter study had 0.41 mm MRD, showing that our younger children achieved better results following training with the described submarine protocol [3]. Also for 8 year-old subjects, Power and colleagues reported mean RMS_abs_ data for two groups of children after exclusion of subjects with more than 1.5 mm mean RMS_abs_. While they do not report how many subjects they excluded based on this criterion, comparison of the datasets after exclusion of the subjects with too much motion showed that the results of 0.64±0.37 mm mean RMS_abs_ displacement of our 5- and 6-year-old children were as good as the results of 0.51±0.29 mm and 0.70±0.31 mm in the 8.5±1 year-old and the 8.8±0.7 year-old groups, respectively. Thus, despite the 2-year difference in age, the motion metrics of the 5- and 6-year-old children following training with the described protocol, are similar or better compared to the results of 8-year-old children reported in the rfMRI literature.

### 4. Importance of motion assessment in children

Both the DTI and rfMRI data show that the motion present in the scans varies substantially between children. When assessing the motion present in scans of young children, it is important to take into account that different methods for assessing motion along with their associated exclusion criteria can lead to different outcomes. As evident by the numerous outliers present in the data (see Figures 1 and 2), conducting appropriate between-group comparisons of motion and using appropriate techniques for assessing and correcting movement on a volume-by-volume basis (e.g., ‘scrubbing’), are essential in studies including scans of such young children [2]. Because relative differences in motion between one group and another can bias the results of motion-sensitive scans such as DTI and fMRI, careful consideration and reporting any differences in motion when comparing subject groups is important [1]–[4]. While assessment of motion is often described in the rfMRI literature, this is still mostly absent in the DTI literature.

## Conclusion

The submarine protocol and data presented in this paper demonstrate the feasibility of conducting relatively long DTI and rfMRI examinations of children as young as 5 years of age, without the use of sedation, or lengthy training procedures. In addition, the feedback given by the parents and the children following the acquisition of the scans confirmed that the MRI scanning sessions were truly considered a positive and fun experience. Aside from averting the possible negative effects of sedation, this approach was also time efficient. Time considerations can be a crucial factor when deciding which approach to take for preparing a child for his/her MRI scan. The pre-scanning preparation phase of approximately 30–45 minutes allowed a more resource-efficient way of managing the MRI scanning of young children compared to routinely using sedation, while still leading to a high success rate. Furthermore, the preparation phase can be conducted by only one motivated specialist in contrast to requiring multiple medical professionals as is necessary when scanning children under sedation. This approach may therefore be useful for scanning of healthy, typically developing children and of clinical populations that otherwise could not be scanned, allowing an in depth investigation of brain mechanisms.

## Supporting Information

Table S1 **Demographics and motion parameters for the 75 children undergoing DTI scanning.**
(DOCX)

Table S2 **Translational (in mm) and rotational (in degrees) DTI motion summaries (mean and standard deviation) of the 67 subjects without extreme motion as defined in Ling and colleagues, 2012.**
(DOCX)

Table S3 **Demographics and motion parameters for the 71 children undergoing rfMRI scanning.**
(DOCX)

Text S1 **Submarine protocol.**
(DOCX)
