# Supplementary material for: Diffusion Tensor Imaging and Resting-State Functional MRI-Scanning in 5- and 6-Year-Old Children: Training Protocol and Motion Assessment
Source: PLoS One. 2014 Apr 9;9(4):e94019. doi: 10.1371/journal.pone.0094019 (PMC3981727; doi:10.1371/journal.pone.0094019)
Supplement: Table S1 — Demographics and motion parameters for the 75 children undergoing DTI scanning. (DOCX) [file pone.0094019.s001.docx]

**Table S1:** Demographics and motion parameters for the 75 children undergoing DTI scanning

| **Subject** | **Age** | **Gender** | **DTI**  **median RMS_abs_ (mm)** | **DTI**  **maximum RMS_abs_ (mm)** | **DTI**  **median RMS_rel_ (mm)** | **DTI**  **maximum RMS_rel_ (mm)** |
| --- | --- | --- | --- | --- | --- | --- |
| 1 | 5.8 | M | 1.92 | 3.86 | 0.17 | 2.16 |
| 2 | 5.9 | M | 1.96 | 6.38 | 0.14 | 3.90 |
| 3 | 5.9 | F | 0.37 | 0.48 | 0.03 | 0.14 |
| 4 | 5.9 | F | 1.15 | 2.85 | 0.20 | 0.69 |
| 5 | 5.9 | F | 0.51 | 0.60 | 0.03 | 0.10 |
| 6 | 5.9 | F | 1.14 | 1.50 | 0.05 | 0.32 |
| 7 | 5.9 | M | 3.04 | 5.51 | 0.51 | 2.69 |
| 8 | 5.9 | M | 0.52 | 0.80 | 0.05 | 0.31 |
| 9 | 5.10 | F | 1.07 | 4.99 | 0.13 | 1.93 |
| 10 | 5.10 | M | 1.10 | 3.82 | 0.34 | 2.68 |
| 11 | 5.10 | F | 0.49 | 1.16 | 0.05 | 0.64 |
| 12 | 5.10 | M | 0.70 | 2.99 | 0.06 | 1.75 |
| 13 | 5.11 | F | 0.41 | 0.67 | 0.04 | 0.14 |
| 14 | 5.11 | M | 0.51 | 0.64 | 0.03 | 0.09 |
| 15 | 5.11 | F | 1.34 | 2.65 | 0.09 | 1.14 |
| 16 | 5.11 | F | 0.86 | 4.01 | 0.08 | 1.99 |
| 17 | 6.0 | F | 0.76 | 4.74 | 0.08 | 4.13 |
| 18 | 6.0 | F | 0.39 | 0.47 | 0.03 | 0.15 |
| 19 | 6.0 | F | 0.55 | 0.77 | 0.04 | 0.22 |
| 20 | 6.0 | M | 0.64 | 1.07 | 0.07 | 0.41 |
| 21 | 6.0 | M | 0.72 | 0.96 | 0.06 | 0.36 |
| 22 | 6.0 | F | 0.46 | 0.88 | 0.06 | 0.44 |
| 23 | 6.0 | M | 0.92 | 2.89 | 0.13 | 0.87 |
| 24 | 6.0 | F | 1.42 | 2.89 | 0.07 | 1.06 |
| 25 | 6.1 | F | 0.40 | 0.81 | 0.04 | 0.19 |
| 26 | 6.1 | F | 0.46 | 0.73 | 0.11 | 0.31 |
| 27 | 6.1 | M | 0.47 | 1.08 | 0.05 | 0.30 |
| 28 | 6.1 | M | 0.54 | 0.85 | 0.06 | 0.17 |
| 29 | 6.1 | M | 0.40 | 0.58 | 0.06 | 0.23 |
| 30 | 6.1 | F | 0.45 | 0.73 | 0.05 | 0.18 |
| 31 | 6.1 | M | 1.60 | 1.72 | 0.05 | 0.48 |
| 32 | 6.1 | M | 0.83 | 1.62 | 0.10 | 0.53 |
| 33 | 6.1 | M | 0.53 | 0.74 | 0.08 | 0.27 |
| 34 | 6.1 | M | 0.75 | 3.46 | 0.11 | 2.46 |
| 35 | 6.2 | F | 1.98 | 13.65 | 0.61 | 5.85 |
| 36 | 6.2 | M | 0.56 | 1.63 | 0.07 | 1.14 |
| 37 | 6.2 | M | 0.50 | 0.66 | 0.04 | 0.12 |
| 38 | 6.2 | F | 0.89 | 1.57 | 0.09 | 0.69 |
| 39 | 6.2 | F | 1.25 | 3.94 | 0.11 | 2.70 |
| 40 | 6.2 | M | 0.80 | 2.77 | 0.12 | 1.16 |
| 41 | 6.2 | M | 0.44 | 1.62 | 0.16 | 1.21 |
| 42 | 6.2 | M | 0.46 | 0.85 | 0.08 | 0.37 |
| 43 | 6.2 | M | 0.42 | 1.16 | 0.10 | 0.90 |
| 44 | 6.3 | F | 0.56 | 0.70 | 0.05 | 0.26 |
| 45 | 6.3 | M | 0.55 | 0.75 | 0.06 | 0.18 |
| 46 | 6.3 | M | 0.44 | 0.77 | 0.05 | 0.20 |
| 47 | 6.3 | M | 1.09 | 2.26 | 0.06 | 1.06 |
| 48 | 6.3 | M | 2.18 | 4.17 | 0.32 | 1.94 |
| 49 | 6.3 | M | 0.34 | 1.72 | 0.06 | 1.17 |
| 50 | 6.4 | F | 0.40 | 0.56 | 0.06 | 0.18 |
| 51 | 6.4 | M | 0.41 | 0.71 | 0.06 | 0.31 |
| 52 | 6.4 | F | 0.64 | 1.64 | 0.08 | 1.03 |
| 53 | 6.4 | M | 1.11 | 3.95 | 0.21 | 2.59 |
| 54 | 6.5 | F | 0.99 | 2.48 | 0.08 | 1.45 |
| 55 | 6.5 | M | 0.54 | 5.99 | 0.07 | 3.80 |
| 56 | 6.5 | F | 0.53 | 0.73 | 0.09 | 0.23 |
| 57 | 6.5 | F | 0.63 | 0.91 | 0.12 | 0.56 |
| 58 | 6.5 | M | 0.59 | 6.28 | 0.08 | 5.07 |
| 59 | 6.5 | M | 0.74 | 3.08 | 0.16 | 1.26 |
| 60 | 6.5 | M | 1.57 | 4.67 | 0.13 | 2.21 |
| 61 | 6.5 | F | 1.38 | 3.98 | 0.07 | 1.64 |
| 62 | 6.5 | M | 0.43 | 0.57 | 0.06 | 0.27 |
| 63 | 6.5 | M | 0.39 | 0.76 | 0.04 | 0.33 |
| 64 | 6.5 | M | 0.63 | 0.80 | 0.06 | 0.27 |
| 65 | 6.5 | M | 0.90 | 1.87 | 0.06 | 0.96 |
| 66 | 6.5 | M | 1.33 | 3.18 | 0.29 | 1.96 |
| 67 | 6.6 | M | 0.51 | 0.83 | 0.08 | 0.43 |
| 68 | 6.6 | F | 0.35 | 0.41 | 0.04 | 0.13 |
| 69 | 6.7 | M | 0.97 | 2.85 | 0.04 | 1.77 |
| 70 | 6.7 | F | 0.73 | 1.56 | 0.13 | 1.12 |
| 71 | 6.7 | M | 0.58 | 1.14 | 0.05 | 0.48 |
| 72 | 6.8 | M | 0.37 | 0.73 | 0.08 | 0.31 |
| 73 | 6.8 | M | 0.37 | 0.58 | 0.04 | 0.22 |
| 74 | 6.8 | M | 1.17 | 2.52 | 0.08 | 1.24 |
| 75 | 6.9 | M | 0.31 | 0.56 | 0.05 | 0.12 |
