# Supplementary material for: Diffusion Tensor Imaging and Resting-State Functional MRI-Scanning in 5- and 6-Year-Old Children: Training Protocol and Motion Assessment
Source: PLoS One. 2014 Apr 9;9(4):e94019. doi: 10.1371/journal.pone.0094019 (PMC3981727; doi:10.1371/journal.pone.0094019)
Supplement: Table S3 — Demographics and motion parameters for the 71 children undergoing rfMRI scanning. (DOCX) [file pone.0094019.s003.docx]

**Table S3:** Demographics and motion parameters for the 71 children undergoing rfMRI scanning

| **Subject** | **Age** | **Gender** | **rfMRI**  **median RMS_abs_ (mm)** | **rfMRI**  **maximum RMS_abs_ (mm)** | **rfMRI**  **median RMS_rel_ (mm)** | **rfMRI**  **maximum RMS_rel_ (mm)** |
| --- | --- | --- | --- | --- | --- | --- |
| 1 | 5.8 | M | 0.36 | 1.17 | 0.02 | 1.06 |
| 3 | 5.9 | F | 0.08 | 0.98 | 0.01 | 0.26 |
| 4 | 5.9 | F | 0.29 | 4.09 | 0.03 | 3.32 |
| 5 | 5.9 | F | 0.41 | 1.59 | 0.03 | 0.67 |
| 6 | 5.9 | F | 0.68 | 4.01 | 0.08 | 3.30 |
| 7 | 5.9 | M | 0.42 | 5.29 | 0.07 | 1.74 |
| 8 | 5.9 | M | 0.08 | 0.24 | 0.01 | 0.17 |
| 9 | 5.10 | F | 0.29 | 3.42 | 0.02 | 2.92 |
| 10 | 5.10 | M | 0.25 | 1.62 | 0.02 | 0.93 |
| 11 | 5.10 | F | 3.06 | 4.48 | 0.05 | 3.08 |
| 12 | 5.10 | M | 0.47 | 5.69 | 0.02 | 4.58 |
| 13 | 5.11 | F | 0.72 | 2.9 | 0.02 | 2.90 |
| 14 | 5.11 | M | 0.27 | 2.8 | 0.02 | 1.34 |
| 15 | 5.11 | F | 1.01 | 3.91 | 0.03 | 2.31 |
| 17 | 6.0 | F | 0.34 | 5.85 | 0.02 | 5.21 |
| 18 | 6.0 | F | 0.38 | 6.43 | 0.05 | 2.65 |
| 19 | 6.0 | F | 0.14 | 3.76 | 0.01 | 2.82 |
| 21 | 6.0 | M | 0.22 | 1.37 | 0.03 | 0.94 |
| 22 | 6.0 | F | 1.14 | 6.59 | 0.03 | 5.12 |
| 23 | 6.0 | M | 2.71 | 3.93 | 0.10 | 2.36 |
| 24 | 6.0 | F | 1.19 | 4.08 | 0.20 | 2.46 |
| 25 | 6.1 | F | 0.6 | 2.85 | 0.03 | 2.30 |
| 26 | 6.1 | F | 0.13 | 0.48 | 0.01 | 0.06 |
| 27 | 6.1 | M | 2.27 | 9.38 | 0.09 | 6.33 |
| 28 | 6.1 | M | 0.47 | 8.9 | 0.07 | 6.05 |
| 29 | 6.1 | M | 0.12 | 0.85 | 0.02 | 0.52 |
| 30 | 6.1 | F | 0.11 | 0.32 | 0.01 | 0.16 |
| 31 | 6.1 | M | 0.42 | 2.94 | 0.05 | 1.87 |
| 32 | 6.1 | M | 0.48 | 4.84 | 0.10 | 3.69 |
| 33 | 6.1 | M | 0.55 | 2.23 | 0.03 | 0.93 |
| 34 | 6.1 | M | 0.87 | 3.25 | 0.04 | 1.62 |
| 35 | 6.2 | F | 3.74 | 13.64 | 0.30 | 7.33 |
| 36 | 6.2 | M | 1.94 | 7.26 | 0.07 | 4.78 |
| 37 | 6.2 | M | 0.32 | 0.69 | 0.01 | 0.28 |
| 38 | 6.2 | F | 0.75 | 5.53 | 0.08 | 5.01 |
| 39 | 6.2 | F | 0.13 | 1.38 | 0.03 | 1.06 |
| 40 | 6.2 | M | 0.39 | 3.54 | 0.03 | 2.08 |
| 41 | 6.2 | M | 1.53 | 5.08 | 0.21 | 4.95 |
| 42 | 6.2 | M | 0.87 | 5.91 | 0.04 | 4.44 |
| 43 | 6.2 | M | 0.39 | 3.5 | 0.03 | 2.38 |
| 44 | 6.3 | F | 0.11 | 0.35 | 0.02 | 0.06 |
| 45 | 6.3 | M | 0.27 | 1.65 | 0.02 | 1.31 |
| 46 | 6.3 | M | 0.1 | 2.35 | 0.02 | 1.69 |
| 47 | 6.3 | M | 0.1 | 1.32 | 0.02 | 0.44 |
| 48 | 6.3 | M | 0.71 | 3.37 | 0.07 | 2.32 |
| 49 | 6.3 | M | 3.54 | 14.8 | 0.34 | 11.23 |
| 50 | 6.4 | F | 0.54 | 4.85 | 0.04 | 3.27 |
| 51 | 6.4 | M | 0.21 | 3.84 | 0.02 | 1.93 |
| 52 | 6.4 | F | 0.56 | 5.14 | 0.03 | 2.44 |
| 53 | 6.4 | M | 1.18 | 4.65 | 0.13 | 3.88 |
| 54 | 6.5 | F | 0.79 | 5.15 | 0.04 | 2.76 |
| 55 | 6.5 | M | 6.2 | 16.22 | 0.22 | 8.66 |
| 56 | 6.5 | F | 1.34 | 2.85 | 0.03 | 1.63 |
| 57 | 6.5 | F | 0.3 | 2.77 | 0.02 | 1.96 |
| 58 | 6.5 | M | 2.22 | 4.59 | 0.20 | 3.01 |
| 59 | 6.5 | M | 0.31 | 1.72 | 0.01 | 1.49 |
| 60 | 6.5 | M | 0.39 | 3.49 | 0.04 | 2.52 |
| 62 | 6.5 | M | 1.14 | 4.8 | 0.06 | 2.84 |
| 63 | 6.5 | M | 0.56 | 1.48 | 0.05 | 0.61 |
| 64 | 6.5 | M | 0.27 | 2.98 | 0.05 | 2.02 |
| 65 | 6.5 | M | 1.17 | 3.62 | 0.03 | 1.79 |
| 66 | 6.5 | M | 3.19 | 8.39 | 0.35 | 4.88 |
| 67 | 6.6 | M | 0.58 | 3.77 | 0.08 | 2.25 |
| 68 | 6.6 | F | 0.12 | 0.98 | 0.01 | 0.43 |
| 69 | 6.7 | M | 0.12 | 4.9 | 0.02 | 3.66 |
| 70 | 6.7 | F | 0.19 | 1.5 | 0.02 | 0.99 |
| 71 | 6.7 | M | 1.03 | 4.97 | 0.02 | 3.07 |
| 72 | 6.8 | M | 0.16 | 0.53 | 0.03 | 0.33 |
| 73 | 6.8 | M | 0.55 | 0.81 | 0.02 | 0.58 |
| 74 | 6.8 | M | 1.64 | 13.59 | 0.14 | 8.17 |
| 75 | 6.9 | M | 0.07 | 0.2 | 0.01 | 0.09 |
