# Supplementary material for: Diffusion Tensor Imaging and Resting-State Functional MRI-Scanning in 5- and 6-Year-Old Children: Training Protocol and Motion Assessment
Source: PLoS One. 2014 Apr 9;9(4):e94019. doi: 10.1371/journal.pone.0094019 (PMC3981727; doi:10.1371/journal.pone.0094019)
Supplement: Text S1 — Submarine protocol. (DOCX) [file pone.0094019.s004.docx]

**Text S1: Submarine protocol**

This protocol aimed to make the MRI scanning session a fun and exciting experience for the child. To achieve this goal, the child was immersed in a story about a submarine adventure and then undertook the scanning session as a submarine ‘captain’. The protocol included three phases, described in detail below. Completion of the full protocol required approximately 30 minutes of pre-hospital preparation, followed by a maximum two hours at the hospital.

**1. Phase 1: Initial contact**

The aim of the initial contact session was two-fold: (i) to ensure the parents were fully aware of the process and were provided with sufficient time to ask any questions they may have; and (ii) to ensure that the child knew what would happen during his/her^1^^[[1]](#footnote-1)^ visit to the hospital.

During the first contact with the parents, in person or by telephone, a clear explanation was given regarding the type and purpose of the scan. The researcher emphasized that the study would be conducted in a child-friendly manner and that the child and parent could ask to stop the session at any time. After the first contact, the parents received an email with an overview of all the practical aspects of the scanning session (e.g., that the children wear comfortable clothes without metal and that they bring the child’s favorite movie^2^ to the scanning session) and in depth information on the purpose of the study. The email further contained a link to two movies featuring a 5-year-old child going through the preparation and scanning phases. One of the movies served as an instruction video for the parents, showing the procedure and the purpose of the research and giving background information about the safety procedures. The other movie was a child-friendly version in which the child met Whally the Whale, a stuffed toy featuring in every step of the scanning procedure. In the movie, Whally explained what would happen on the day of visiting the hospital to receive the scan.

Based on our observations made during the initial contact phase, giving the parents sufficient background information and time to ask questions helped them to feel comfortable and focus on their child during phases 2 and 3. While some of the parents did not think it was necessary to show the video to their children, most of them acknowledged that their children would feel more comfortable knowing what would happen before the hospital visit.

**2. Phase 2: Pre-scanning preparation**

The aims of this phase were to make the child feel at ease with the researcher and to teach the child how to comply with the scanning procedures.

On the day of the scan, the researcher met the child in the entrance hall of the hospital together with Whally the Whale. The preparation took approximately 45 minutes and was performed in a child-friendly location.

We observed that, as most of the children had already seen the movie at home, they recognized the researcher and Whally the Whale as the ‘movie stars’. They seemed to be honored to meet the characters from the movie and this seemed to make them feel at ease. The walk towards the MRI facilities was an ideal moment for an informal conversation with the child, their parents and siblings. This conversation allowed the researcher to observe important information about the child’s behavior which helped to decide on how to approach the child during the preparation phase.

We deemed it important to invest a sufficient amount of time in the preparation phase as it gave the child the time to adjust to the researcher and the environment. However, if necessary, this phase could be completed within half an hour with cooperative children.

Upon arrival in the preparation room, the researcher first gave the parents the necessary forms to complete. Next, she explained to the child that he/she would participate in an exciting adventure with a submarine. During the ‘ride’ in the submarine, beautiful pictures of his/her head would be taken. The researcher emphasized that not all children were allowed to dive in the submarine. Only the brave children who could complete all the tasks necessary to earn a diploma of Submarine Captain would be allowed to see the submarine and dive in it. The researcher showed the diploma as well as the chart with the 6 tasks that had to be completed. The number of tasks was counted and the child learned that he/she could choose a sticker after each completed task. Following completion of the six tasks, he/she would receive the diploma with his/her name printed on it.

The six tasks to be completed by the child were chosen to allow him/her to practice all the potentially difficult aspects of undergoing an MRI scan. By breaking these into different steps, all practiced in a fun and active way in a child-friendly environment, we wanted to make the scanning a great experience instead of potentially overwhelming and scary. A concise description of the six tasks is provided below. These tasks can be expanded based on the child’s reaction and time needed for them to adjust to the setting.

*Task 1 – Popping bubbles:*

Purpose: the first task aimed to build a positive interaction between the child and the researcher and make the child feel at ease.

Material: bubble maker

Procedure: the researcher asked the child if he/she knew what happens when a fish is breathing under water. In many movies or pictures, bubbles escape from the mouth of the fish. The researcher explained that she wanted to check if the child was afraid of bubbles as he/she may see some while diving in the submarine. The researcher then showed the bubble maker to the child and said she would verify if the child dared to pop the bubbles. The child was encouraged to run around in the room and pop the bubbles. The researcher started by blowing only a few bubbles and made the task more difficult and interactive according to the child’s skills. Next, the child could check whether the researcher was afraid of bubbles. The child blew bubbles and let the researcher run around to pop the bubbles. The aim of this was to build rapport with the child and ensure he/she was enthusiastic to continue with the other tasks. The task ended by telling the child that he/she could take the bubble maker home and choose the first sticker for his/her sticker chart.

*Task 2 – Good and blurry pictures:*

Purpose: this task was used to explain to the child that the pictures of his/her brain would be blurry if he/she moved while lying in the scanner.

Material: booklet of sharp and blurry pictures of a submarine, a child, a cat, brains, etc. Some pictures were only a little blurred, others were very blurry, and some only partially showed the object. The booklet started with a sharp picture of the object, followed by blurry pictures of the same object to ensure that the difference was obvious to the child.

Procedure: the researcher explained once more that the submarine was actually a giant photo camera and that pictures of the child’s head would be taken while diving in the submarine. These were not ordinary pictures but cool pictures of what was inside his/her head. Did he/she know what is inside his/her head? Brains! The picture of the researcher’s brain in a decorated frame on the table was shown (see task 3) and a conversation was initiated about the funny shapes of the brain. The researcher then asked the child whether he/she had any experience in taking pictures of his/her parents, siblings or pets. Did he/she know what happened when they moved? The picture got all blurry! The researcher showed the child a book of pictures made by Whally the Whale and asked if he/she could see which pictures were good and which pictures were taken during movement. The researcher emphasized that the child would have to restrict his/her movements in the submarine to allow nice brain pictures to be taken.

*Task 3 – Picture frame:*

Purpose: the third task was designed to maximize the cooperation of the child in the scanner by visualizing a reward.

Material: picture frames in different colors, foam letters of the child’s name, and other water-related frame decorations.

Procedure: The researcher reminded the child of the previous task: if he/she laid very still, nice pictures of his/her brain could be taken. The researcher showed the framed picture of her brain again. If she can take sharp pictures, the child can take a picture of his/her brain home! This is very cool and special as no other children have such a picture! The researcher asked the child if he/she wanted to make a special frame for his/her picture and let him choose a frame color. The researcher then gave the child the letters of his/her name, which he/she could glue onto the frame together with additional decorative items to personalize his/her frame. The decorated empty frame was placed next to the researcher’s frame and it was explained once more that the child’s picture would be put in the frame if he/she cooperated well in the scanner.

*Task 4 – Candy on nose:*

Purpose: to instruct the child about how to lie still for a long period of time.

Material: bed, candy fishes

Procedure: now that the child knew he/she had to refrain from moving in the submarine, the researcher would check if he/she was able to do this. For this task, the child had to climb up on the bed and lay down on his/her back. The researcher said she had a special trick for testing if he/she moved his/her head: placing a candy fish on his/her nose! If the candy didn’t fall off while the researcher was counting to ten, the child would be allowed to eat it! The researcher started by putting a little candy fish at the junction of the child’s nose and forehead. When the candy was placed in this position, he/she could move a little without the candy falling off. The researcher then slowly counted to ten and let the child eat the candy. The researcher then made the task ‘much more difficult’ by putting the candy on the tip of the child’s nose.

After completion of this task, we observed that some children felt more confident about laying still. The ‘candy on the nose’ task could be used as a reminder once the child is in the scanner and gave the child the opportunity to ask any questions he/she might have, e.g. can I breathe while I lay still?

*Task 5 – Bucket talk:*

Purpose: to allow the child to get used to the head coil and practice answering questions with words (instead of nodding or shaking his/her head).

Material: colored bucket with a hole cut out for the eyes and a funny face drawn on it.

Procedure: The researcher explained that we all have to wear helmets when we do special things, such as riding a motorcycle. This is also the case in the submarine. While in the submarine, the child would still be able to speak to the researcher through the helmet and headphones. Because the child should not move his/her head to avoid blurry pictures, he/she would have to answer using words. This is similar to speaking on the telephone.

The researcher tested whether the child could do this by putting on the funny bucket helmet. Once the child put on the helmet, the researcher asked questions about his/her plans for the day, preferred food, favorite movie, etc. and reminded the child to answer using words and keep his/her head as still as possible.

*Task 6 – Tunnel crawl:*

Purpose: this task let the child experience the feeling of wearing earplugs and earphones, and of being in a small tunnel.

Material: earplugs, earphones and toy tunnel

Procedure: The researcher explained that the engine of the submarine makes a lot of noise, like an airplane. To make the child feel comfortable and to ensure he/she could listen to his/her favorite movie in the scanner, the researcher would give him earplugs and headphones. The parent accompanying the child would also have to wear these. The researcher then showed the child the earplugs and inserted them in his/her ear. Next, the headphones were placed on his/her head. To illustrate that the child could still hear, the researcher gave him instructions while wearing the earplugs/earphones. This allowed the child to get used to the feeling of wearing these. Next, the child had to perform exercises while wearing the hearing protection. The researcher asked the child to walk in a straight line, walk backwards towards the wall, jump back like a frog, etc. The researcher gave a demonstration of all these tasks to be certain that the child understood the exercise and to further strengthen rapport with the child. The task was finished by introducing a ‘very difficult task’ in which the child had to crawl through a toy tunnel holding Whally the Whale. In the submarine space would be limited and the child would also have to go into a tunnel! First, the child had to crawl through the tunnel on hands and knees. The task ended by asking him to move through the tunnel on his/her back, as this simulated the feeling of going in the tunnel of the MRI machine.

**3. Scanning session**

Following completion of the six tasks, the child had earned all his/her stickers. Once the sticker chart was full, the researcher showed the diploma, ticked all the boxes and put a big, important signature on the page. The researcher expressed that she was very proud of the child and announced that he/she was now allowed to see the submarine! By using this approach, we aimed to let the child see the visit to the submarine as a reward. We preferred to proceed directly to the scanner room as some children got very excited and active during a stay in the waiting area. We also checked that the child used the bathroom before going into the scanner room.

After finishing the safety checks, the child entered the scanner room with one parent. As Whally the Whale loved diving in the submarine, he/she always accompanied the child in the scanner. In our experience, the child felt more comfortable holding something while being in the scanner. Holding Whally also prevented him from holding the alarm bell and accidently pressing it. The scanner room was decorated in a child-friendly manner. A large cardboard submarine was placed in front of the scanner and cardboard fishes, shells, seahorses, etc. were used to hide any appliances that could remind the child of doctors and hospitals. The researcher gave the child the necessary time to explore the scanner room and attracted his/her attention to the television screen at the other end of the MRI tunnel where the child’s movie was playing. In our setup, the movie was shown upside down on the television screen. The researcher asked the child whether he/she was good at watching a movie upside down and then showed the coil and the mirror and explained the trick that would allow him to see the movie correctly. As the child was the only one with a diploma, only he/she could wear the helmet with the mirror! So mum/dad and the researcher would have to look at the video upside-down! Next, the child sat down on the scanner’s bed, the researchers handed a set of earplugs to the parent and inserted the child’s earplugs. Once the child was comfortably seated on the bed, the researcher explained in detail what would happen in the submarine: the submarine would make multiple ‘rides’, the engine could make different noises during different rides and could sometimes shake a little, similar to driving in a car. The researcher also showed where she would sit behind the computer and demonstrated that she could see the child and his/her parents at all times. The child received the alarms bell which was introduced as the submarine’s horn. The researcher emphasized that the horn could only be used when something was wrong. He/she could not press it when the movie reached a funny scene or if his/her little toe itched! If he/she would honk the horn, the researcher would stop the engine noise to talk to him but this also meant that all the rides would have to start all over again, which we did not want. When the child lay down on the submarine’s bed, the researcher emphasized that this was a really cool bed because it could go up all by itself. She bet his/her bed at home is not able to do that! The researcher maintained eye contact and a cheerful expression while the bed was moving upwards to ensure the child saw it as a positive experience. Next, the researcher gently fixated the child’s head by placing padding between the head and the coil to help him keep it still. The researcher installed the headcoil, together with the mirror, while telling the child he/she had the important task of checking if he/she could see the video and whether it was still upside-down or not. The researcher ensured the child could see their parents and could feel his/her mum/dad holding his/her leg. The researcher then slid the bed into the scanner while constantly talking to the child and having the child focus on whether he/she could still see the movie. Once the child was comfortable, the researcher went to the computer room and started talking through the headphones. In the computer room, she asked the child if he/she could hear her and if he/she would like her to restart the movie from the beginning. This enabled the researcher to check if the child was responding properly in the scanner. The researcher then explained that the submarine would leave for its first ‘ride’ and wished the child lots of fun! The researcher talked to the child between each of the scans to keep him/her motivated, telling him/her when a longer scan came and reminding him/her to lay very still. Children who moved during the reference scans usually laid still when the researcher told them she could see excessive movement on her computer screen.

After completion of the scan session, the children received a small toy and the framed picture of their brain to take home.

1. For ease of reading, we will only use double terminology to refer to the child and use the term ‘researcher’, together with female terminology to refer to the person preparing the child for his/her scan.

   ^2^ This should not be a very funny movie to avoid inducing laughter – and thus motion - during the scan session. [↑](#footnote-ref-1)
